# Facile construction of ZIF-94/PAN nanofiber by electrospinning for the removal of Co(II) from wastewater

**DOI:** 10.1038/s41598-023-50796-9

**Published:** 2024-01-03

**Authors:** Cong Yin, Yinyin Peng, Hongjiang Li, Guang Yang, Guoyuan Yuan

**Affiliations:** 1 Xi’an Research Institute of Hi-Tech, Xi’an, 710025 People’s Republic of China; 2 Chongqing University of Science and Technology, Chongqing, 401331 People’s Republic of China

**Keywords:** Chemistry, Polymer synthesis

## Abstract

This study aimed to synthesize a novel nanofiber adsorbent based on metal–organic frameworks (MOFs), ZIF-94-PAN, by incorporating ZIF-94 into polyacrylonitrile (PAN) through electrospinning. The investigation of the adsorption characteristics of ZIF-94-PAN for cobalt ions was undertaken, yielding findings that suggest an optimum ZIF-94 loading content within the ZIF-94-PAN composite of 8%. The adsorption experiments revealed that, under pH 8.3 and 298 K, ZIF-94-PAN-8% attained cobalt ion equilibrium adsorption (139.08 mg/g). Additionally, the adsorption kinetics of cobalt ions exhibited conformity with the pseudo-second-order model, whereas adherence to the Freundlich isotherm model indicated a non-homogeneous, endothermic process. XPS analysis unveiled that the adsorption mechanism was characterized by the coordination of nitrogen and oxygen atoms within ZIF-94-PAN with cobalt ions. This study effectively addressed the challenges of separating and recovering MOFs adsorbents by fabricating them as nanofibers. The remarkable adsorption performance and stability of the ZIF-94-PAN nanofibers highlight their potential for removing cobalt-contaminated wastewater.

## Introduction

Cobalt is a typical toxic heavy metal element, classified as a 2B carcinogen, along with cobalt compounds^1^. Its entry into water magnifies its impact on the food chain and accumulates in human organs, posing chronic toxicity risks. Consequently, the removal of cobalt from water is essential.

Currently, there are several treatment methods for cobalt ions, including adsorption^2–4^, membrane separation^5^, electrolysis^6^, chemical precipitation^7^, and ion exchange^8^. Of these methods, adsorption stands as the prevailing choice in practical applications owing to its inherent simplicity and notable efficacy. The selection of adsorbents is crucial for the adsorption method. Carbon adsorbents^9^, mineral adsorbents^10^, polymer adsorbents^11^, and biological adsorbents^12^ are commonly employed. However, the high cost and difficulty in regenerating traditional adsorbents make their usage economically unfavorable. Consequently, there is an escalating demand for the development of novel, cost-effective adsorbents to address this exigency. This has resulted in the emergence of a hot research topic in this field—the development of new and efficient adsorbents.

Metal–organic framework (MOF) materials represent a class of porous materials synthesized via the chemical coordination of metal ions with organic bridging ligands^13^. These materials exhibit numerous merits, encompassing a notable specific surface area, substantial pore volume, commendable chemical stability, tunable pore dimensions, and a multitude of functional sites. These properties make MOFs materials highly suitable for heavy metal adsorption, such as cobalt.

In recent years, the preparation of MOFs nanofibers (NFs) using electrospinning has gained significant attention^14^. This is due to the limitations of powdered MOFs, such as tube clogging during adsorption and separation processes^15,16^. Additionally, particle aggregation can lead to decreased adsorption capacity, and recycling powdered MOFs for reuse becomes challenging. Conversely, electrospinning is a mild, simple, and cost-effective method for producing NFs^17^. By utilizing electrospinning, MOFs powders can be transformed into self-supporting flexible MOFs NFs embedded within polymer NFs^18–23^. These NFs offer a substantial specific surface area, elevated porosity, excellent mechanical resilience, and favorable permeability, making them ideal for supporting MOFs crystals. Importantly, MOFs in the form of fibers can be easily removed from the reaction system without the need for centrifugation or other separation operations. This simplifies the material’s reuse and enhances its practicality.

This paper focuses on the preparation of MOFs NFs for the separation of cobalt ions using electrospinning, where polyacrylonitrile (PAN) serves as the substrate and ZIF-94 acts as the filler. The prepared MOFs NFs undergo comprehensive characterization using techniques. The performance of the MOFs NFs in the separation of cobalt ions is evaluated through batch experiments, assessing their separation ability, stability, and recoverability. Furthermore, an exploration of the underlying cobalt adsorption mechanism of the MOFs NFs is conducted. These experiments provide evidence of the effectiveness of MOFs NFs in removing cobalt from aqueous solutions and their potential for practical applications.

## Experimental methods

### Chemicals and experimental instruments

The main raw materials including polyacrylonitrile (PAN), 4-methylimidazole-5-carboxaldehyde, taurine (TAUR), zinc acetate dihydrate (Zn(CH_3_COO)_2_·2H_2_O), tetrahydrofuran (THF), cobalt nitrate hexahydrate (Co(NO_3_)_2_·6H_2_O), methanol, and N,N-dimethylformamide (DMF) were procured from Shanghai Aladdin Biochemical Science and Technology Co.

The adsorption experiments were performed using an air-bath thermostatic shaking chamber (HZQ-F160, China). The quantification of metal ion concentrations after adsorption was ascertained employing ICP-MS, specifically the EXPEC 7000 instrument. MOFs NFs were fabricated utilizing an electrospinning apparatus (YFSP-T, China). The crystal structure analysis of the adsorbed materials was conducted through XRD (smartlab9, Japan), and the surface morphology and elemental distribution were observed using SEM (Thermo Fisher Scientific Apreo 2C) and EDS (X-Max, Oxford, UK). The surface functional groups were analyzed using FT-IR (Thermo Scientific FT-IR Nicolet 6700, Japan). The specific surface area and pore characteristics were accomplished through N_2_ adsorption–desorption analysis (Micro for TriStar II Plus 3030, USA). The chemical valence states of surface elements were examined through XPS (Thermo Fisher Scientific, Escalab 250Xi+ , USA) before and after adsorption.

### Synthesis of ZIF-94

ZIF-94 synthesis was carried out in accordance with the methodology described in the existing literature^24^. 6.33 g of 4-methylimidazole-5-carbaldehyde was dissolved in 48 mL of THF. A solution of 6.34 g of Zn(CH_3_COO)_2_·2H_2_O in 24 mL of methanol was prepared, followed by the addition of this zinc acetate solution to the 4-methylimidazole-5-carbaldehyde solution with vigorous agitation. After 24 h of uninterrupted agitation at room temperature, the resultant product underwent centrifugation and subsequent washing with methanol. Subsequently, it was subjected to an additional 24 h drying period at room temperature.

### Synthesis of ZIF-94-PAN

**Figure 1:**
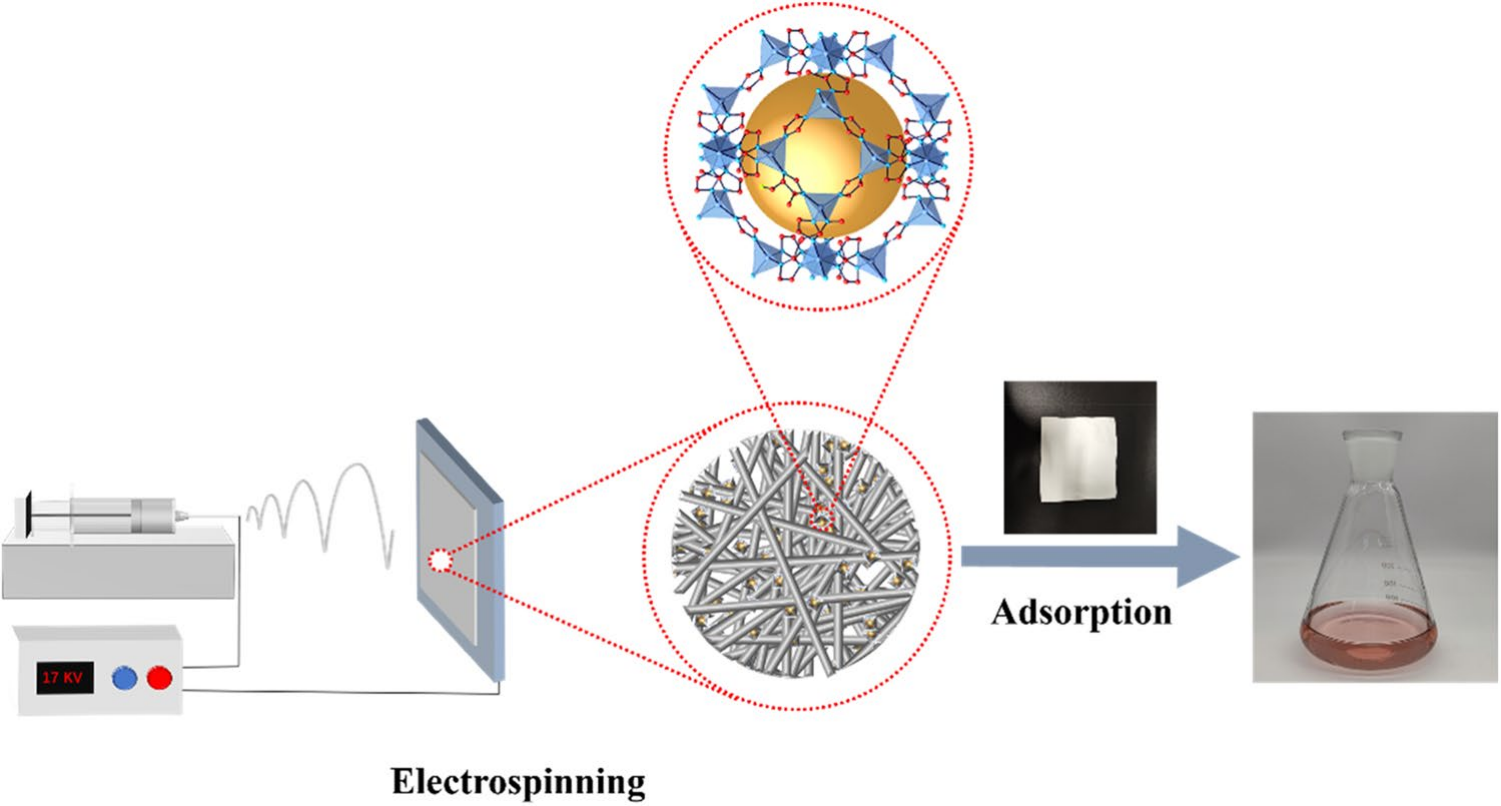
Schematic illustration of an experiment.

0.6 g PAN, 0.5 g PVP K30, and a specified quantity of ZIF-94 were homogeneously dispersed in 5 mL of DMF, stirred for 4 h, and then left to remove air bubbles. Subsequently, 4 mL of the above solution was used for electrospinning. The voltage was controlled at 17 kV, while the suspension exhibited a flow rate of 0.0018 mm/s. After 4 h of spinning, the ZIF-94 NFs were obtained. The NFs were then subjected to vacuum drying at 60 °C for a duration of 6 h and immediately transferred to hot deionized water to remove PVP K30, resulting in ZIF-94-PAN-*x* (with different doping amounts, *x* = 4%, 8%, and 12%) (Fig. 1).

### Adsorption experiments of Co(II) by ZIF-94-PAN

The adsorption capacity of ZIF-94-PAN for Co(II) was tested by adsorbing Co(II) in water. 0.0010 g of ZIF-94-PAN was meticulously weighed and introduced into 50 mL of Co(II) solution, spanning various concentrations. Following this, the mixture was then transferred to an air-bath thermostatic shaking chamber for the experiment. The effect of ZIF-94-PAN on the separation performance of Co(II) was investigated under different parameters (pH, time, concentration, temperature, etc.), and the adsorption amount was calculated employing Eq. (1).

1 $$ q_{t} = \frac{{\left( {C_{0} - C_{{\text{t}}} } \right) \times V}}{m} $$

here, *q*_*t*_ represents the amount adsorbed at time t (mg/g), *C*_*0*_ and *C*_*t*_ (mg/L) denote the concentrations of cobalt ions at the initial and time *t*, *V* signifies the volume (L) of the solution employed in the adsorption experiments, and *m* stands for the mass of the MOFs NFs (g).

## Results and discussion

### Characterization

**Figure 2:**
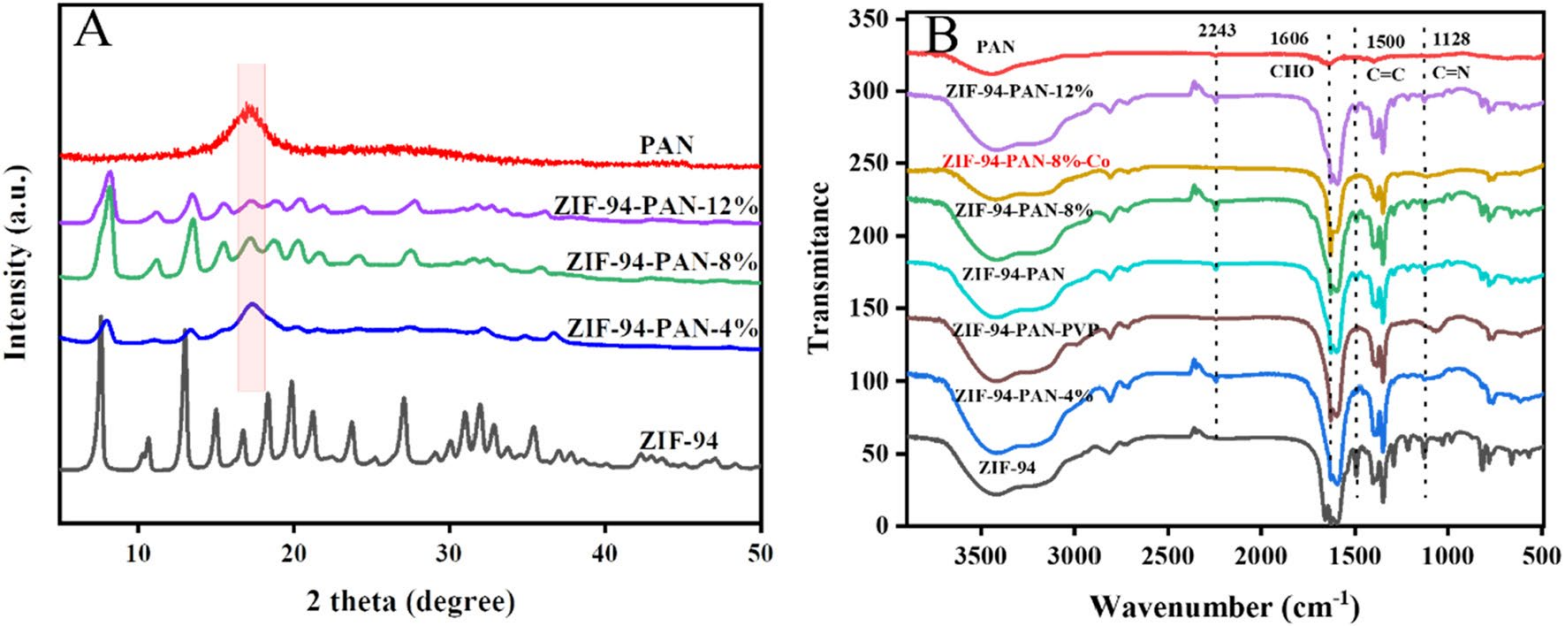
XRD patterns (**A**) and FT-IR spectra (**B**) of PAN, ZIF-94, and ZIF-94-PAN.

The crystallographic characteristics of the prepared ZIF-94 and ZIF-94-PAN were determined using X-ray diffractograms collected by XRD, employing Cu-Kα radiation and spanning a scanning range (5°–60°). Analysis of Fig. 2A reveals that the broad peaks appearing at 2θ = 16.9° are indicative of the amorphous structural nature of PAN^25^. The presence of characteristic diffraction peaks of both ZIF-94 and PAN at various contents of ZIF-94-PAN provides preliminary evidence for the successful preparation of the materials^26^. It also suggests that ZIF-94 maintains its intact crystal structure in the prepared composites. The surface functional groups of the synthesized materials were examined via FT-IR. The FT-IR spectra of ZIF-94, ZIF-94-PAN-8%-Co and different contents of ZIF-94-PAN (Fig. 2B), featuring similar spectra with main bands located at 1606 cm^-1^ corresponding to the aldehyde group (–CHO)^27^, whereas the aldehyde group peak is weakened after cobalt adsorption by ZIF-94-PAN-8%, and the C=N peak at 1128 cm^-1^ is weakened after adsorption can indicate the presence of C=C bonds during cobalt adsorption. The C=N peak at 1128 cm^-1^ is weakened after adsorption which indicates that cobalt chelates with aldehyde group and N upon adsorption. The band at 1500 cm^-1^ indicates the presence of C=C bonding^28^. One of the peaks observed at 2243 cm^-1^ can be ascribed to the stretching vibrational mode associated with the C≡N bond in PAN^29^. The peaks at these major spectral bands were gradually enhanced with the increase of MOFs, further demonstrating the successful composite of ZIF-94 into PAN NFs.

**Figure 3:**
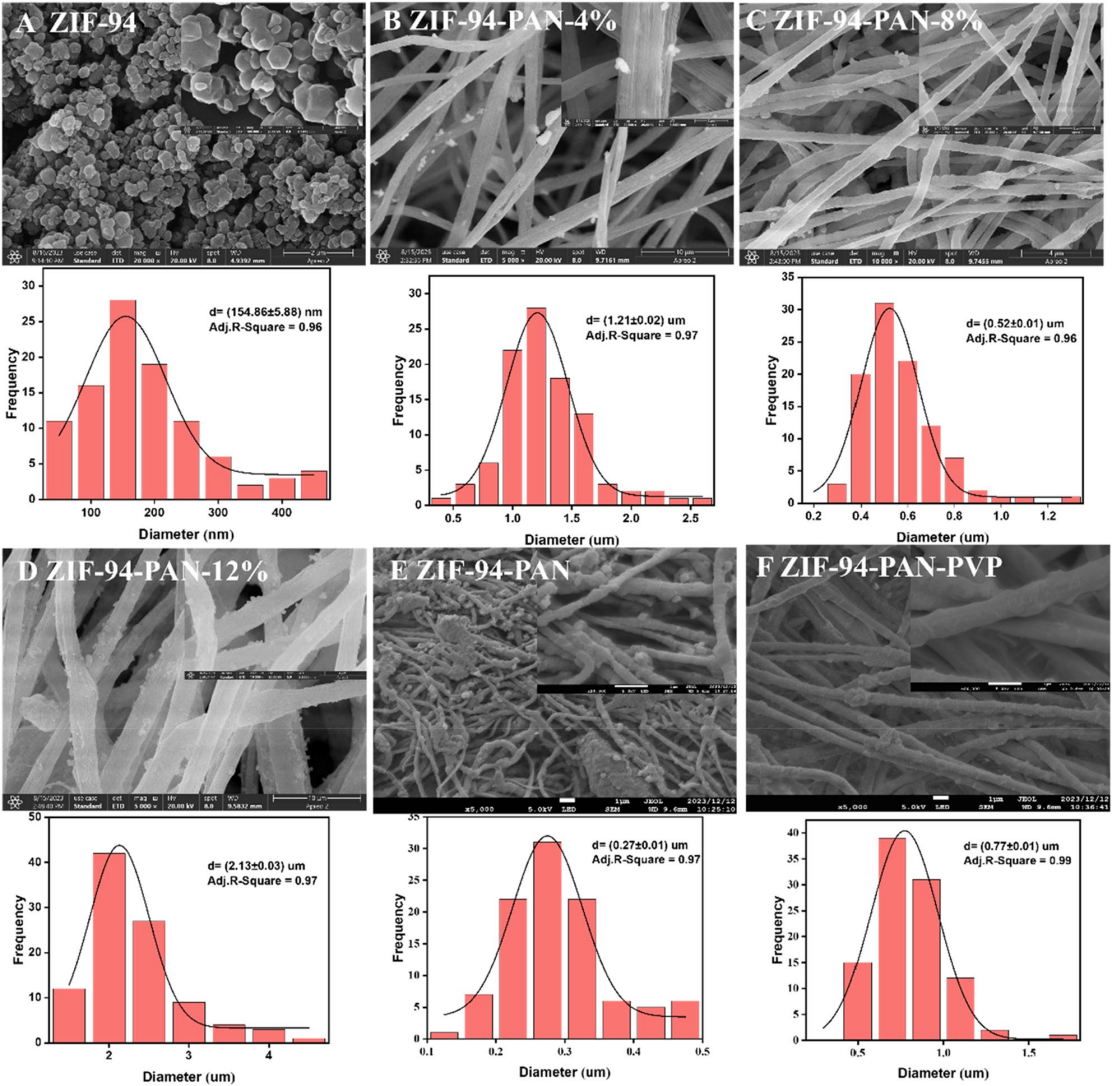
SEM image of (**A**) ZIF-94, (**B**) ZIF-94-PAN-4%, (**C**) ZIF-94-PAN-8%, (**D**) ZIF-94-PAN-12%, (**E**) ZIF-94-PAN, and (**F**) ZIF-94-PAN-PVP.

The SEM images depicted in Fig. 3A–D illustrate both ZIF-94 and ZIF-94-PAN NFs, which underwent a grinding process to ensure proper particle size adaptation of ZIF-94 to PAN during blending. Notably, Fig. 3B–D provides insights into the morphology and structure of ZIF-94-PAN NFs with varying ZIF-94 contents. Analyzing the images, it is evident that the composite fibers with an 8% ZIF-94 content exhibit the most homogeneous distribution. In contrast, in the case of composite fibers containing 4% ZIF-94, the distribution of ZIF-94 on the surface is sporadic, leading to a diminished adsorption capacity. Moreover, composite fibers with a 12% ZIF-94 content clearly demonstrate reduced dispersion and noticeable agglomeration of ZIF-94. This agglomeration leads to pore blockage and a consequent reduction in adsorption, which aligns with the findings from the adsorption experiments. Given that the NFs consisted of two polymers, one of which was water soluble (PVP), the potential hydrolysis of the fibers was investigated. To ensure the stability of the fibers and to prevent any secondary contamination, PAN and 8%-ZIF-94 were spun (ZIF-94-PAN), PAN, PVP, and 8%-ZIF-94 (ZIF-94-PAN-PVP), and fibers eluting PVP (ZIF-94-PAN-8%) were subjected to comparative SEM and FTIR analyses (Figs. 2B, 3E,F). It was found that the eluted NFs remained stable. The decrease in the diameter of NFs after elution of ZIF-94-PAN-PVP is due to the elution of PVP.

**Figure 4:**
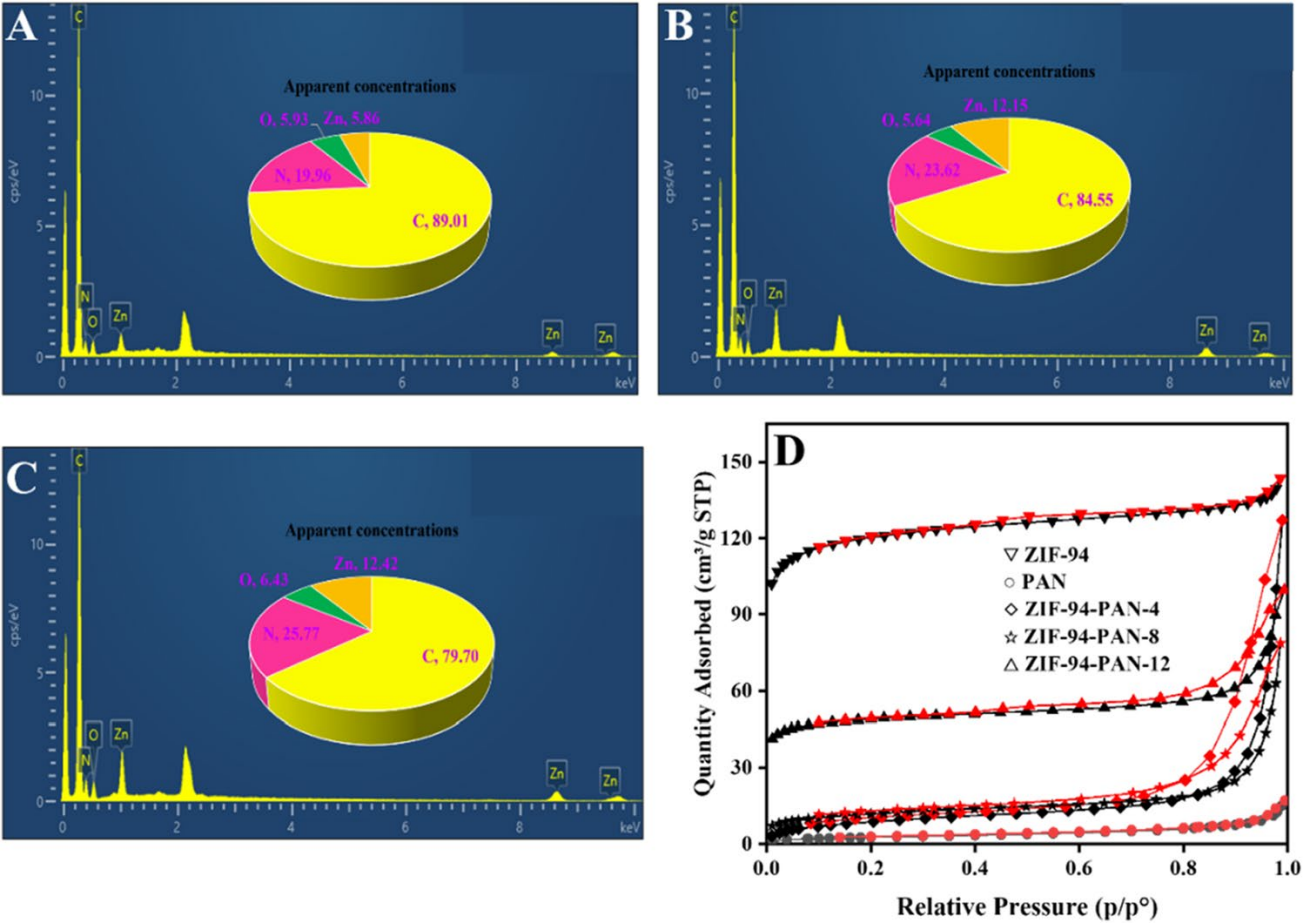
The EDS energy spectra of (**A**) ZIF-94-PAN-4%, (**B**) ZIF-94-PAN-8% and (**C**) ZIF-94-PAN-12%. (**D**) N_2_ adsorption–desorption isotherms.

EDS analysis (Fig. 4A–C) provides further evidence of the composite formation between ZIF-94 and PAN NFs. As anticipated, all four elements, namely C, O, N, and Zn, were detected on the surface of the composites. Additionally, the apparent concentration of Zn increases with the higher ZIF-94 content. This observation supports the successful incorporation of ZIF-94 into the PAN NFs.

The specific surface area, pore size, and pore volume of PAN, ZIF-94, and ZIF-94-PAN were determined using BET analysis to examine the impact of different contents of ZIF-94 on the properties of the NFs (Fig. 4D). Table 1 presents the obtained results. PAN exhibited a mere 0.7 m^2^/g, while ZIF-94 exhibited a significantly higher value of 470.3 m^2^/g. As the ZIF-94 content increased from 4 to 8%, there was observed a concurrent augmentation in both the specific surface area and pore volume. Nonetheless, with a subsequent increase in ZIF-94 content to 12%, a concurrent decrease in both specific surface area and pore volume was observed. This decline can be attributed to the inhomogeneous dispersion and agglomeration caused by the increased ZIF-94 content. Consequently, the polymer molecules obstructed the pore structure, which aligns with the observations from the SEM analysis.

**Table 1:** The pore structure data of materials.

Materials	Surface area (m^2^/g)	Pore volume (cm^3^/g)	Pore diameter (nm)
PAN	0.7	0.001	1.834
ZIF-94	470.3	0.186	1.889
ZIF-94-PAN-4%	33.5	0.006	23.368
ZIF-94-PAN-8%	190.9	0.070	3.189
ZIF-94-PAN-12%	43.1	0.013	11.307

### Adsorption experiment

#### Effect of pH

**Figure 5:**
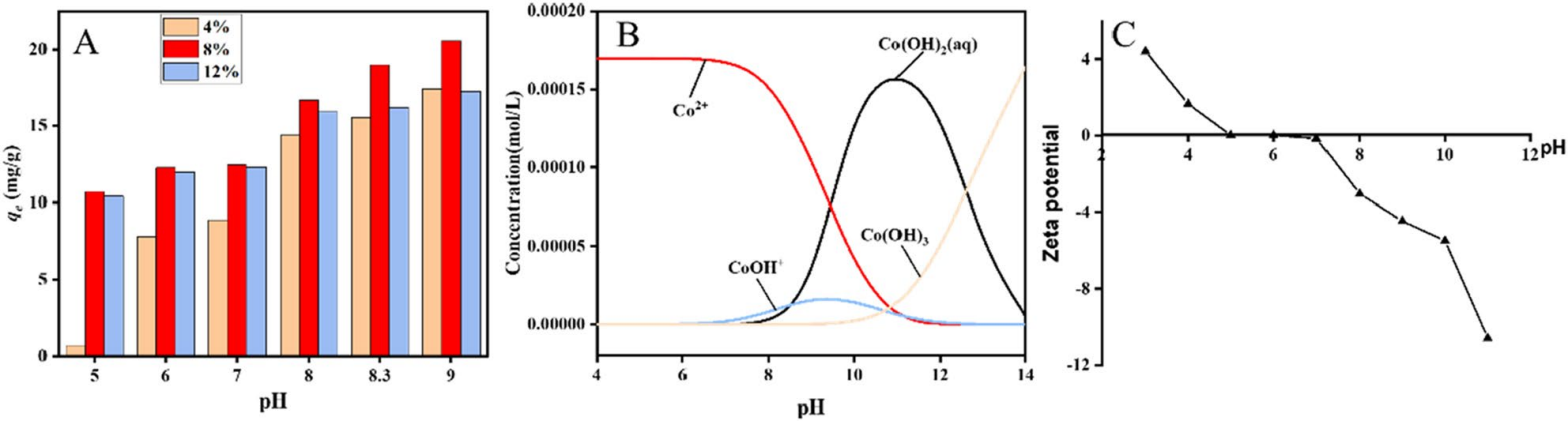
The impact of (**A**) pH on adsorption performance, and (**B**) several major forms of cobalt ions at different pH. Zeta potential of ZIF-94-PAN-8% (**C**).

Figure 5A illustrates the effect of pH on the cobalt ion adsorption performance of ZIF-94-PAN. The figure reveals that within the pH range of 5–9, there is a notable enhancement in the adsorption of cobalt ions by ZIF-94-PAN with an increase in the solution's pH. However, when the pH reaches 9, precipitation of cobalt ions occurs, as indicated by the analysis conducted using visual MINTEQ software (Fig. 5B)^30^. After deducting the amount of precipitation, the actual adsorption amount decreases. The maximum adsorption amount, 18.99 mg/g, is achieved under pH conditions of 8.3. This occurrence can be attributed to the prevalence of acidic conditions, fostering an elevated concentration of H^+^ ions, thereby engendering competitive interactions with cobalt ions for adsorption sites. H^+^ ions is higher than that the diffusion rate of Co(II) ions, making acidic conditions unfavorable for Co(II) adsorption. As pH increases, a reduction in the concentration of H^+^ ions occur, concomitantly leading to an augmentation in the availability of unoccupied adsorption sites, thereby enhancing the adsorption capacity. Conversely, when Co(II) exists in a strongly alkaline environment, it takes the form of Co(OH)_2_ in solution, which hinders adsorption. Moreover, in Fig. 5C, it can be used to see that the material has no electrostatic repulsion with cobalt when the surface presents a negative charge at pH > 6. In addition, the adsorption capacity of ZIF-94-PAN-8% (18.99 mg/g) is significantly greater than that of ZIF-94-PAN-4% (15.53 mg/g) and ZIF-94-PAN-12% (16.18 mg/g). Initially, increasing the doping amount leads to increased adsorption due to the increased presence of MOFs. However, when the doping amount reaches 12%, MOFs start to agglomerate, which causing polymer molecules to clog the pore structure of ZIF-94. As a result, cobalt ions are unable to enter the pores, leading to a decrease in adsorption amount. Based on these findings, ZIF-94-PAN-8% was selected for subsequent research under the conditions of pH 8.3, adsorbent mass of 10 mg, time of 24 h, initial concentration of 10 ppm, and volume of 50 mL.

#### Effect of contact time

**Figure 6:**
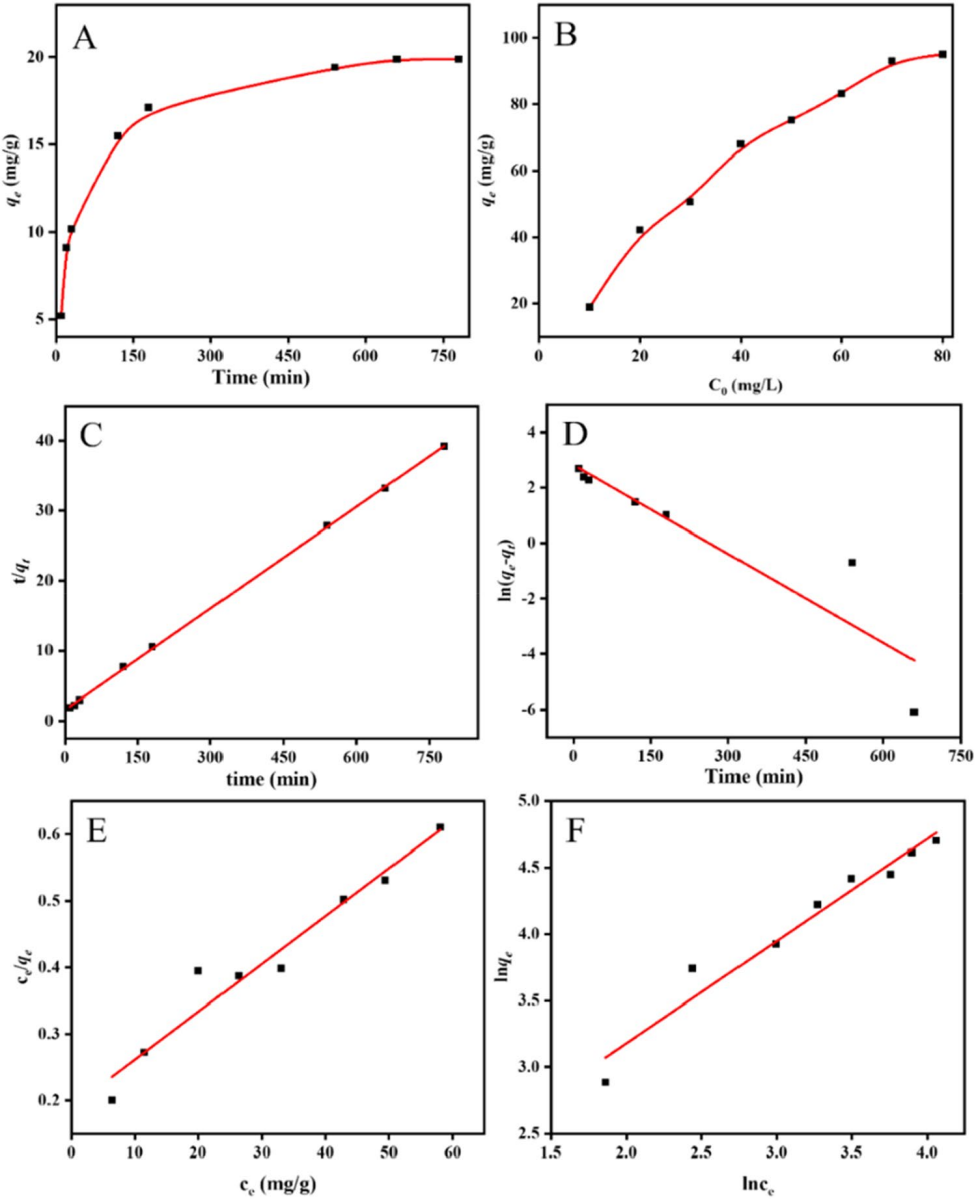
Impact of (**A**) contact time. and (**B**) cobalt ions concentration. The fitting curves for (**C**) pseudo-first-order, (**D**) pseudo-second-order kinetic model, (**E**) Langmuir model, and (**F**) Freundlich model.

The impact of contact time on the cobalt ion adsorption efficacy of ZIF-94-PAN-8% is presented in Fig. 6A. The contact time range was 10–780 min. The surface area of the adsorbent provides a place for the adsorption process and increases the probability of the adsorbent interacting with cobalt. The adsorption initially showed a rapid increase with increasing contact time. However, at 9 h, the curve starts to flatten out, indicating a slower rate of adsorption. This can be attributed to the availability of initial adsorption sites on the material. As the time of adsorption progresses, a significant portion of these sites becomes engaged, leading to a decrement in the adsorption rate. To delve deeper into the adsorption mechanism, Pseudo-first-order and pseudo-second-order models were used to investigate the mechanism. The equations for these models are provided below^31,32^:

2 $$ \ln \left( {q_{e} - q_{t} } \right) = \ln q_{e} - k_{1} t $$

3 $$ \frac{t}{{q_{t} }} = \frac{1}{{k_{2} q_{e}^{2} }} + \frac{t}{{q_{e} }} $$

Here, *k*_*1*_ and *k*_*2*_ denote the rate constants associated with the pseudo-first-order and pseudo-second-order models, while *q*_*e*_ and *q*_*t*_ signify the adsorbed quantity at equilibrium and at a given adsorption time *t* (mg/g).

The results of the fitting procedure are visually represented in Fig. 6C,D. The calculated values are presented in Table 2, indicating that the linear correlation coefficient for the pseudo-second-order model (R^2^ = 0.999) surpasses that of the pseudo-first-order model (R^2^ = 0.922). Moreover, the theoretical maximum adsorption capacity of 20.70 mg/g is closer to the experimental adsorption amount of 19.88 mg/g. These findings imply the predominance of the pseudo-second-order kinetic model, where the rate-determining step in the adsorption process is predominantly controlled by chemical adsorption. In other words, the adsorption process is primarily a combination of ZIF-94-PAN-8% and Co(II) through sharing or exchanging electronic valence bonds.

**Table 2:** The kinetic model parameters.

Pseudo-first-order			Pseudo-second-order		
*q*_*e*_	*k*_*1*_	R^2^	*q*_*e*_	*k*_*2*_	R^2^
16.62	1.07 × 10^–2^	0.922	20.70	1.48 × 10^–3^	0.999

#### Effect of initial cobalt ions concentration

Figure 6B demonstrates the influence of the initial concentration of Co(II) solution on the adsorption efficacy of ZIF-94-PAN-8%. The initial concentration range was 10–80 ppm. The graph depicts a gradual rise in the adsorption of Co(II) ions by ZIF-94-PAN-8% as the initial concentration of cobalt ions increases. However, when the initial Co(II) concentration reaches 70 mg/L, the Co(II) adsorption reaches a plateau (94.97 mg/g), indicating adsorption equilibrium as all the adsorption sites on the adsorbent are fully occupied. To elucidate the Co(II) adsorption mechanism, Langmuir (Eq. 4) and Freundlich (Eq. 5) adsorption isotherm models were employed. The equations are as follows^33,34^:

4 $$ \frac{{c_{e} }}{{q_{e} }} = \frac{{c_{e} }}{{q_{m} }} + \frac{1}{{q_{m} k_{L} }} $$

5 $$ \ln q_{e} = \ln K_{F} + \frac{1}{n}\ln c_{e} $$

Here, *q*_*e*_ and *q*_*m*_ are the equilibrium and maximum theoretical adsorption of Co(II) (mg g^-1^), respectively. *c*_*e*_ represents the equilibrium concentration of Co(II) (mg/L). *K*_*L*_ represents the Langmuir adsorption equilibrium constant (L mg^-1^), while *K*_*F*_ (mg/g (L/mg^1/n^) and *n* represent the Freundlich constants linked to adsorption capacity and adsorption strength, respectively.

The fitted plots are visualized in Fig. 6E,F. The parameter values are presented in Table 3. Notably, the enhanced agreement between the experimental data and the Freundlich isotherm model, as indicated by the elevated correlation coefficient (R^2^ = 0.978), implies non-homogeneity in the adsorption process.

**Table 3:** The isotherm model parameters.

Langmuir			Freundlich		
*q*_*m*_	*K*_*L*_	R^2^	*K*_*F*_	*n*	R^2^
139.08	0.0380	0.952	5.18	1.303	0.978

#### Effect of temperature

**Figure 7:**
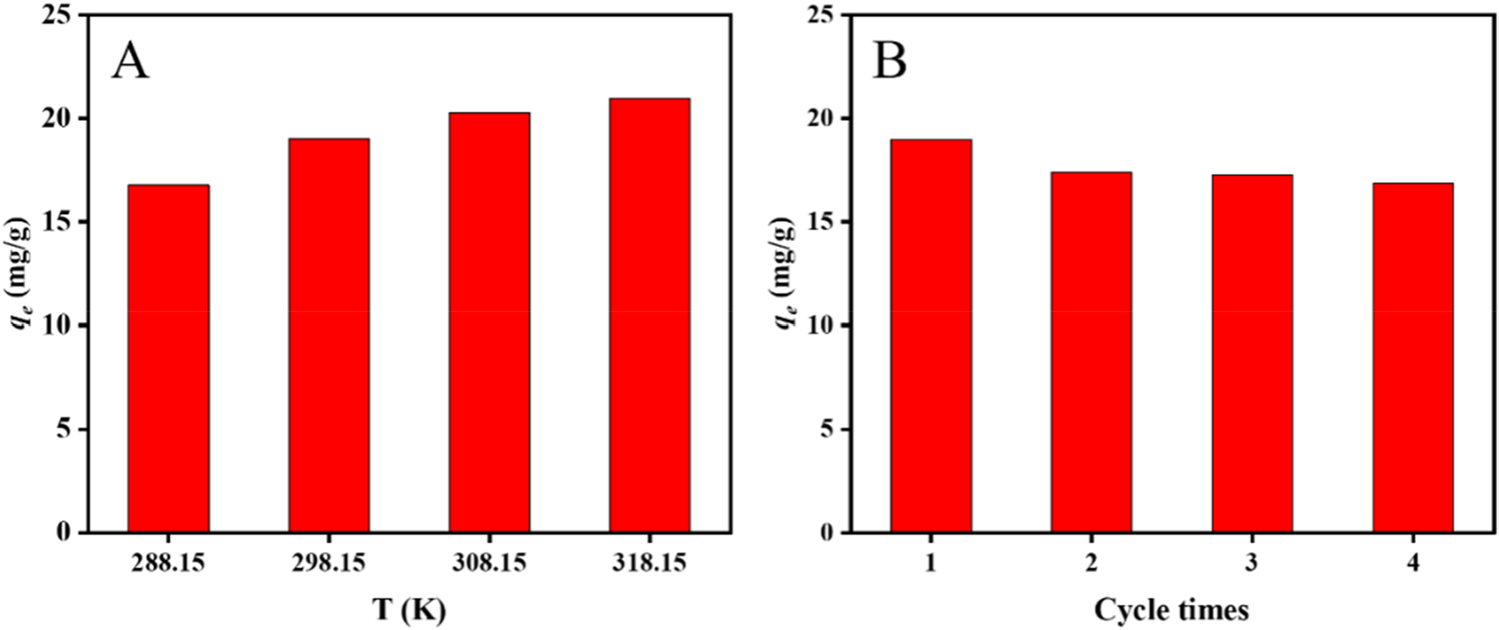
Effect of (**A**) temperature on adsorption performance and (**B**) recycling.

In order to assess how temperature affects the adsorption performance of ZIF-94-PAN-8%, adsorption experiments were carried out at various temperatures, as depicted in Fig. 7A. The temperature range is 288.15–318.15 K. As the temperature increases, the adsorption of cobalt ions by the adsorbent material shows an upward trend. This is primarily due to the reversible nature of the adsorption process, encompassing both adsorption and desorption phases. The rate of cobalt ion adsorption is accelerated with temperature elevation. To gain deeper insight into the adsorption process, thermodynamic properties were investigated through the calculation of thermodynamic parameters. The thermodynamic enthalpy change (*ΔH*^*0*^), entropy change (*ΔS*^*0*^), and Gibbs free energy change (*ΔG*^*0*^) were determined using the following formulas. The thermodynamic parameters were obtained by linear plotting according to Eq. (8)^35^:

6 $$ K_{{\text{d}}} = q_{e} {/}c_{e} $$

7 $$ \vartriangle G = - RT\ln K_{d} $$

8 $$ \ln K_{d} = \frac{{\Delta S^{0} }}{R} - \frac{{\Delta H^{0} }}{RT} $$

Here, *K*_*d*_ indicates the thermodynamic equilibrium constant, *R* denotes the universal gas constant (8.314 J/(mol K)), and *T* denotes the absolute temperature expressed in Kelvin (K).

Table 4 presents the thermodynamic data. The negative value of *ΔG*^*0*^ decreases with increasing temperature, indicating that the adsorption of Co(II) on ZIF-94-PAN-8% is a spontaneous process and that an increase in temperature favors the adsorption reaction. *ΔH*^*0*^ have positive values, suggesting that the adsorption process is endothermic.

**Table 4:** Adsorption thermodynamic parameters.

Temperature(K)	ln*K*_*d*_	*ΔG*^*0*^(J/mol)	*ΔH*^*0*^(kJ/mol)	*ΔS*^*0*^(J/mol K)
288.15	3.47 × 10^–3^	− 8.31	102.3	489.2
298.15	3.35 × 10^–3^	− 8.30		
308.15	3.25 × 10^–3^	− 8.33		
318.15	3.13 × 10^–3^	− 8.28		

#### Study on cycle stability

To explore the cyclic regeneration ability of ZIF-94-PAN-8% in practical use, 0.1 mol/L nitric acid was used as the desorbent after adsorption. As shown in the Fig. 7B, ZIF-94-PAN-8% maintained stable adsorption performance after 4 times of use, indicating that this adsorbent has unlimited potential for cobalt ion separation and enrichment.

### Adsorption mechanism

**Figure 8:**
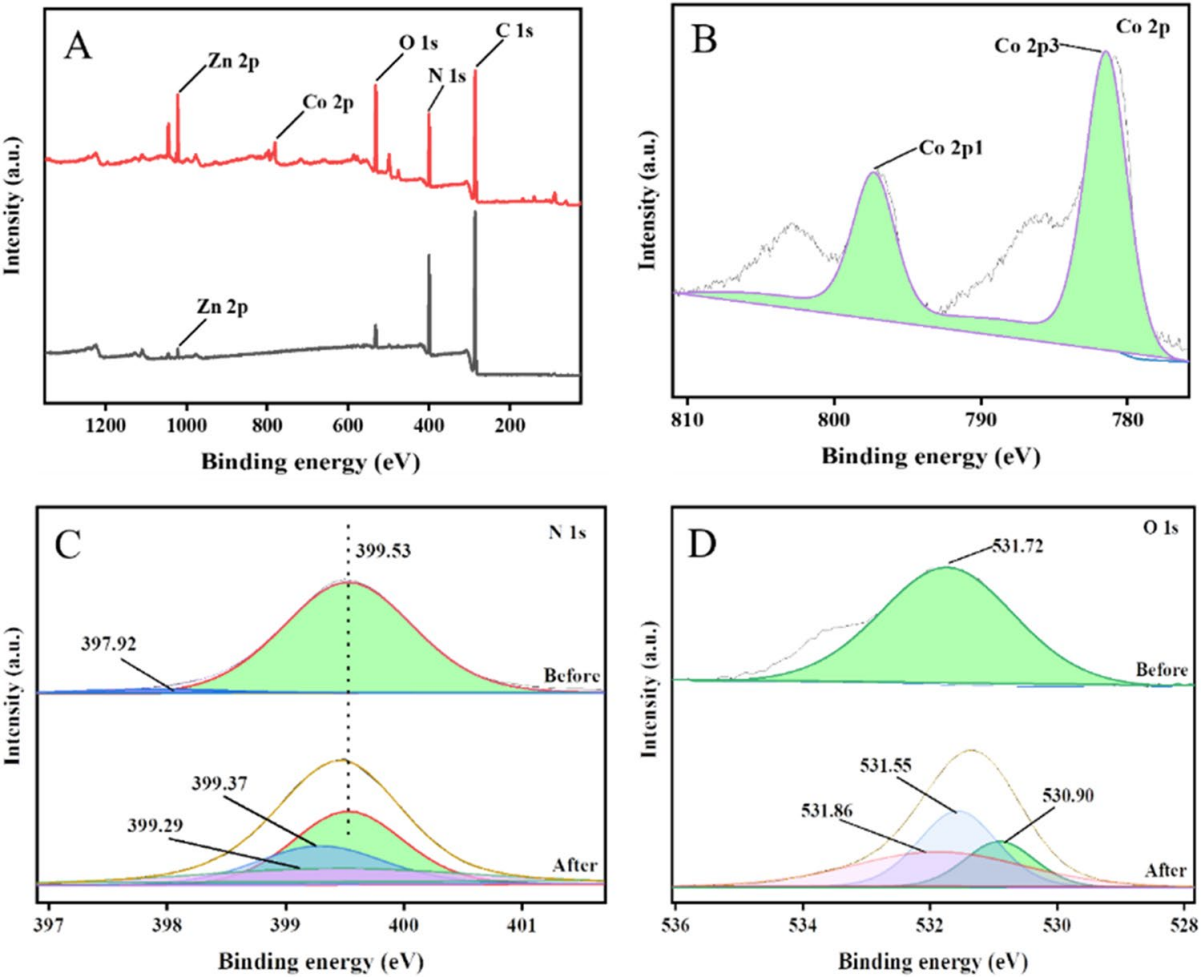
XPS scan full spectrum (**A**), Co 2p spectrum (**B**), N 1 s spectrum (**C**), and O 1 s spectrum (**D**).

The elucidation of the adsorption mechanism of Co(II) on ZIF-94-PAN-8% was explained using XPS test. From the full spectra before and after adsorption (Fig. 8A), it can be observed that all of them have the presence of elements C, N, O and Zn. However, following adsorption, Co(II) is observed in the full spectrum, confirming the successful adsorption of Co(II) onto ZIF-94-PAN-8%. The Co 2p spectrum showed characteristic peaks corresponding to Co 2p^1^ and Co 2p^3^ (Fig. 8B), with energies measured at 781.43 and 797.27 eV, respectively. As shown in the Fig. 8C, before and after adsorption of one of the N on the imidazole ring peaked at 399.53 eV, with little change, but after the adsorption of cobalt, the binding energy of the other C=N on the imidazole ring shifted from 397.92 to 399.37 eV^36^, and the C=N binding energy of the imidazole ring shifted from 397.92 to 399.37 eV, and the cobalt binding energy of the imidazole ring moved from 397.92 to 399.37 eV. This is due to the coordination of cobalt with N. An additional peak at 399.29 eV following adsorption is ascribed to the addition of the pH buffer taurine -NH_2_. After cobalt adsorption, the binding energy of the O 1 s spectrum decreases, which may be due to the coordination of cobalt with oxygen as well. The newly appeared peaks 531.86 eV and 531.55 eV can be ascribed to the S=O and O–H functionalities of taurine, respectively (Fig. 8D) ^37^. Drawing from the preceding discourse, the cobalt adsorption mechanism on ZIF-94-PAN-8% can be ascribed to the coordination between the C=N of the imidazolium ring and the oxygen group of –CHO with Co(II).

## Conclusion

In this paper, the electrospinning technique was utilized to successfully fabricate ZIF-94-PAN composite nanofibers with ZIF-94 loadings of 4%, 8%, and 12%, employing PAN as a matrix. These fabricated nanofibers derived from MOFs demonstrate high effectiveness as adsorbents for cobalt removal from wastewater, effectively mitigating the challenge of recycling powdered MOFs. The most favorable ZIF-94 loading was identified at 8%, displaying a notable cobalt adsorption capacity of 139.08 mg/g. Based on our investigations, adsorption kinetics follow pseudo-second-order, while isotherm adheres to Freundlich model. Notably, following four consecutive cycles, the adsorption efficiency was found to maintain 95% of the initial uptake. These findings underscore the substantial promise of ZIF-94-PAN-8% nanofibers for practical applications in the realm of wastewater treatment.

## Data Availability

All data generated or analysed during this study are included in this published article.
